# Supplementary material for: Basic Guide for Approaching Drug Delivery with Extracellular Vesicles
Source: Int J Mol Sci. 2024 Sep 27;25(19):10401. doi: 10.3390/ijms251910401 (PMC11476574; doi:10.3390/ijms251910401)
Supplement: Supplementary file 1 [file ijms-25-10401-s001.zip › ijms-3213992-supplementary.pdf]

NCT Number	Study Title	Exosome source	Modificaitons	Study URL	Interventions
NCT06571799	Study Evaluating the Efficacy and Safety of BENEV Exosome Regenerative Complex+ for Self-perceived Thinning Hair	Undefined Stem cells		https://clinicaltrials.gov/study/NCT06571799	OTHER: BENEV Exosome Regenerative Complex+
NCT06568653	Human Placenta Mesenchymal Stem Cells Derived Exosomes Injection for Treatment of Complex Anal Fistula	MSC		https://clinicaltrials.gov/study/NCT06568653	PROCEDURE: exosome|PROCEDURE: routine conventional fistulotomy
NCT06543667	Limbal Stem Cell Derived Exosome (LSC-Exo) Eye Drop for Treatment of Dry Eye	Limbal Stem Cells		https://clinicaltrials.gov/study/NCT06543667	BIOLOGICAL: Limbal Stem Cell Derived Exosomes Eye Drop
NCT06539273	Exosome Treatment in Androgenetic Alopecia	MSC		https://clinicaltrials.gov/study/NCT06539273	"DRUG: Exosome Complex, RNA"
NCT06536712	Effects of Exosome Adminstration in Preventing Early Leakage	MSC		https://clinicaltrials.gov/study/NCT06536712	BIOLOGICAL: Mesenchymal Stem Cells Derived Exosomes|OTHER: Placebo

	in Rectal Cancer Patients Undergoing Low Anterior Resection				
NCT06492798	Effectiveness and Safety of Mesenchymal Stem Cell Therapy in Long COVID Patients	MSC		https://clinicaltrials.gov/study/NCT06492798	DRUG: umbilical cord mesenchymal stem cell
NCT06482541	Efficacy and Safety Of AGE ZEROв„ў EXOSOMES To Treat Men and Women With Androgenetic Alopecia	MSC		https://clinicaltrials.gov/study/NCT06482541	COMBINATION_PRODUCT: 5 billion exosomes and saline solution microneedling|COMBINATION_PRODUCT: 50 billion exosomes and saline solution microneedling
NCT06466850	Mesenchymal Stem Cells Derived Exosomes in Osteoarthritis Patients	MSC		https://clinicaltrials.gov/study/NCT06466850	BIOLOGICAL: Exosome
NCT06463132	Phase 1b Clinical Trial to Evaluate PEP and EUFLEXXA for Knee Osteoarthritis (KOA)	Unspecified	Unmodified	https://clinicaltrials.gov/study/NCT06463132	COMBINATION_PRODUCT: PEP/Euflexxa|DRUG: PEP
NCT06431152	Intra-articular Injection of UC-MSC Exosome in	MSC		https://clinicaltrials.gov/study/NCT06431152	BIOLOGICAL: UC-MSC sEV

	Knee Osteoarthritis				
NCT06429033	Purified Exosome Product (PEP) Injected Into the Hypodermis	Unspecified	Unmodified	https://clinicaltrials.gov/study/NCT06429033	DRUG: Purified Exosome Product (PEP)
NCT06391307	The Role of Mesenchymal Stem Cell and Exosome in Treating Pilonidal Sinus Disease in Children	MSC		https://clinicaltrials.gov/study/NCT06391307	OTHER: Crystallized phenol|BIOLOGICAL: Crystallized phenol + Exosome|BIOLOGICAL: Crystallized phenol + Stem Cell|BIOLOGICAL: Crystallized phenol + Exosome + Stem Cell
NCT06319287	"Phase 2a Multi-Center Prospective Randomized Trial to Evaluate the Safety & Efficacy of Topical PEP-TISSEEL for Diabetic Foot Ulcers (DFU)"	Unspecified	Unmodified	https://clinicaltrials.gov/study/NCT06319287	BIOLOGICAL: PEP (Purified Exosome Product) / TISSEEL
NCT06279039	Clinical Observation of Exosomes in Patients After Q-switched Laser Surgery	Unspecified		https://clinicaltrials.gov/study/NCT06279039	DRUG: Exosome liquid dressing
NCT06245746	UCMSC-Exo for Chemotherapy-induced	MSC		https://clinicaltrials.gov/study/NCT06245746	BIOLOGICAL: umbilical cord derived mesenchymal stem cells exosomes (UCMSC-Exo)

	Myelosuppression in Acute Myeloid Leukemia				
NCT06239207	Efficacy and Safety of Exosomes Versus Platelet Rich Plasma in Patients of Androgenetic Alopecia	Unspecified stem cells		https://clinicaltrials.gov/study/NCT06239207	BIOLOGICAL: Exosomes GFC CELL EXO SCALP KIT (Leuco Exo 97%)｜BIOLOGICAL: Platelet Rich Plasma
NCT06221787	Stem Cell Derived Exosomes in the Treatment of Melasma and Its Percutaneous Penetration	MSC		https://clinicaltrials.gov/study/NCT06221787	PROCEDURE: 1565 nm non-ablative fractional laser combined with normal saline｜PROCEDURE: microneedles combined with hUCMSC-Exos｜PROCEDURE: 1565 nm non-ablative fractional laser combined with hUCMSC-Exos｜PROCEDURE: PBASM combined with hUCMSC-Exos
NCT06138210	The Effect of GD-iExo-003 in Acute Ischemic Stroke	iPSC		https://clinicaltrials.gov/study/NCT06138210	DRUG: exosomes derived from human induced pluripotent stem cell for injection｜DRUG: a placebo of exosomes derived from human induced pluripotent stem cell for injection
NCT06072794	A Proof of Concept Study to Evaluate Exosomes From Human Mesenchymal Stem Cells in Women With Premature Ovarian	MSC		https://clinicaltrials.gov/study/NCT06072794	DRUG: VL-PX10

	Insufficiency (POI)				
NCT05969717	Induced Pluripotent Stem Cell Derived Exosomes for the Treatment of Atopic Dermatitis	iPSC		https://clinicaltrials.gov/study/NCT05969717	DRUG: GD-iExo-001|OTHER: normal saline
NCT05947747	Safety and Efficacy of EXO-CD24 in Preventing Clinical Deterioration in Patients With Mild-Moderate ARDS		CD-24 overexpression https://www.ncbi.nlm.nih.gov/pmc/articles/PMC10779124/	https://clinicaltrials.gov/study/NCT05947747	DRUG: EXO-CD24|OTHER: Placebo
NCT05886205	Induced Pluripotent Stem Cell Derived Exosomes Nasal Drops for the Treatment of Refractory Focal Epilepsy	iPSC		https://clinicaltrials.gov/study/NCT05886205	DRUG: iPSC-Exos
NCT05871463	Effect of Mesenchymal Stem Cells-derived Exosomes in Decompensated Liver Cirrhosis	MSC		https://clinicaltrials.gov/study/NCT05871463	BIOLOGICAL: MSC-derived exosomes

NCT05843799	A Study to Evaluate the Safety and Tolerability of ILB-202		I-KB loaded https://www.iliasbio.com/pipeline/srIkB.php	https://clinicaltrials.gov/study/NCT05843799	DRUG: ILB-202 | DRUG: Placebo
NCT05813379	Mesenchymal Stem Cells Derived Exosomes in Skin Rejuvenation	MSC		https://clinicaltrials.gov/study/NCT05813379	COMBINATION_PRODUCT: exosime injection
NCT05808400	Safety and Efficacy of Umbilical Cord Mesenchymal Stem Cell Exosomes in Treating Chronic Cough After COVID-19	MSC		https://clinicaltrials.gov/study/NCT05808400	BIOLOGICAL: MSC-derived exosomes
NCT05787288	A Clinical Study on Safety and Effectiveness of Mesenchymal Stem Cell Exosomes for the Treatment of COVID-19.	MSC		https://clinicaltrials.gov/study/NCT05787288	BIOLOGICAL: Extracellular Vesicles from Mesenchymal Stem Cells
NCT05738629	Safety and Efficacy of Pluripotent Stem Cell-derived Mesenchymal Stem Cell Exosome (PSC-	MSC		https://clinicaltrials.gov/study/NCT05738629	DRUG: Pluripotent Stem Cell-derived Mesenchymal Stem Cell Exosome (PSC-MSC-Exo) Eye Drops

	MSC-Exo) Eye Drops Treatment for Dry Eye Diseases Post Refractive Surgery and Associated With Blepharospasm				
NCT05669144	Co-transplantation of Mesenchymal Stem Cell Derived Exosomes and Autologous Mitochondria for Patients Candidate for CABG Surgery	MSC		https://clinicaltrials.gov/study/NCT05669144	BIOLOGICAL: mitochondria and MSC-derived exosomes
NCT05658094	Exosome Effect on Prevention of Hairloss	MSC		https://clinicaltrials.gov/study/NCT05658094	DEVICE: Exosome
NCT05559177	"An Open Dose-escalation Clinical Study of Chimeric Exosomal Tumor Vaccines for Recurrent or Metastatic Bladder Cancer"	exosomes from APCs induced with tumor antigens from monocytes		https://clinicaltrials.gov/study/NCT05559177	BIOLOGICAL: Chimeric exosomal tumor vaccines
NCT05523011	Safety and Tolerability	MSC		https://clinicaltrials.gov/study/NCT05523011	DRUG: Exosome ointment

	Study of MSC Exosome Ointment				
NCT05499156	Safety of Injection of Placental Mesenchymal Stem Cell Derived Exosomes for Treatment of Resistant Perianal Fistula in Crohn's Patients	MSC		https://clinicaltrials.gov/study/NCT05499156	OTHER: placenal MSC derived exosomes
NCT05490173	The Pilot Experimental Study of the Neuroprotective Effects of Exosomes in Extremely Low Birth Weight Infants	MSC		https://clinicaltrials.gov/study/NCT05490173	OTHER: Exosomes derived from mesenchymal stromal cells (MSCs)
NCT05475418	Pilot Study of Human Adipose Tissue Derived Exosomes Promoting Wound Healing	Adipose tissue		https://clinicaltrials.gov/study/NCT05475418	PROCEDURE: Adipose tissue derived exosomes
NCT05413148	The Effect of Stem Cells and Stem Cell Exosomes on Visual Functions in	MSC		https://clinicaltrials.gov/study/NCT05413148	BIOLOGICAL: Subtenon injection of Wharton jelly derived mesenchymal stem cells|BIOLOGICAL: Subtenon injection of Wharton jelly derived mesenchymal stem cell exosomes|OTHER: Placebo

	Patients With Retinitis Pigmentosa				
NCT05402748	Safety and Efficacy of Injection of Human Placenta Mesenchymal Stem Cells Derived Exosomes for Treatment of Complex Anal Fistula	MSC		https://clinicaltrials.gov/study/NCT05402748	OTHER: placenta-MSCs derived exosomes
NCT05387278	Safety and Effectiveness of Placental Derived Exosomes and Umbilical Cord Mesenchymal Stem Cells in Moderate to Severe Acute Respiratory Distress Syndrome (ARDS) Associated With the Novel Corona Virus Infection (COVID-19)	MSC		https://clinicaltrials.gov/study/NCT05387278	DRUG: EV-Pure™ and WJ-Pure™|DRUG: Placebo
NCT05375604	A Study of exoASO-STAT6 (CDK-004) in Patients		ASO-stat loaded PTGFRN expressing https://www.cancer.gov/publications/dictionaries/cancer-drug/def/aso-stat6-loaded-ptgfrn-expressing-exosomes-cdk-004	https://clinicaltrials.gov/study/NCT05375604	DRUG: CDK-004

	With Advanced Hepatocellular Carcinoma (HCC) and Patients With Liver Metastases From EIther Primary Gastric Cancer or Colorectal Cancer (CRC)				
NCT05354141	Extracellular Vesicle Treatment for Acute Respiratory Distress Syndrome (ARDS) (EXTINGUISH ARDS)	MSC		https://clinicaltrials.gov/study/NCT05354141	BIOLOGICAL: ExoFlo丨OTHER: Intravenous normal saline
NCT05261360	Clinical Efficacy of Exosome in Degenerative Meniscal Injury	MSC		https://clinicaltrials.gov/study/NCT05261360	DRUG: SF-MSC-EX丨DRUG: SF-MSC
NCT05228899	Zofin to Treat COVID-19 Long Haulers	Amniotic fluid	Unmodied	https://clinicaltrials.gov/study/NCT05228899	DRUG: Zofin丨OTHER: Placebo
NCT05216562	Efficacy and Safety of EXOSOME-MSC Therapy to Reduce Hyper-inflammation	MSC		https://clinicaltrials.gov/study/NCT05216562	DRUG: Exosome-MSC Intravenous injection丨DRUG: Placebo Intravenous Injection丨DRUG: COVID-19 Standard Treatment

	In Moderate COVID-19 Patients				
NCT05060107	Intra-articular Injection of MSC-derived Exosomes in Knee Osteoarthritis (ExoOA-1)	MSC		https://clinicaltrials.gov/study/NCT05060107	BIOLOGICAL: Exosomes (sEVs)
NCT05043181	Exosome-based Nanoplatform for Ldlr mRNA Delivery in FH	MSC	Loaded with LDLR mRNA	https://clinicaltrials.gov/study/NCT05043181	BIOLOGICAL: Low Density Lipoprotein Receptor mRNA Exosomes
NCT04969172	"A Phase II Randomized, Double-blind, Placebo-controlled Study to Evaluate the Safety and Efficacy of Exosomes Overexpressing CD24 to Prevent Clinical Deterioration in Patients With Moderate or Severe COVID-19 Infection"		CD-24 overexpression https://www.ncbi.nlm.nih.gov/pmc/articles/PMC10779124/	https://clinicaltrials.gov/study/NCT04969172	DRUG: Exosomes overexpressing CD24
NCT04902183	Safety and Efficacy of Exosomes Overexpressing		CD-24 overexpression https://www.ncbi.nlm.nih.gov/pmc/articles/PMC10779124/	https://clinicaltrials.gov/study/NCT04902183	DRUG: CovenD24

	CD24 in Two Doses for Patients With Moderate or Severe COVID-19				
NCT04879810	Plant Exosomes +/- Curcumin to Abrogate Symptoms of Inflammatory Bowel Disease	Plant		https://clinicaltrials.gov/study/NCT04879810	"PROCEDURE: Sigmoidoscopy and biopsy, blood work"
NCT04849429	Intra-discal Injection of Platelet-rich Plasma (PRP) Enriched With Exosomes in Chronic Low Back Pain	Platelet rich plasma	Unmodified	https://clinicaltrials.gov/study/NCT04849429	BIOLOGICAL: Platelet rich plasma (PRP) with exosomes|DRUG: Normal Saline
NCT04798716	The Use of Exosomes for the Treatment of Acute Respiratory Distress Syndrome or Novel Coronavirus Pneumonia Caused by COVID-19	MSC		https://clinicaltrials.gov/study/NCT04798716	DRUG: MSC-exosomes delivered intravenously every other day on an escalating dose: (2:4:8)|DRUG: MSC-exosomes delivered intravenously every other day on an escalating dose (8:4:8)|DRUG: MSC-exosomes delivered intravenously every other day (8:8:8)
NCT04747574	Evaluation of the Safety of CD24-Exosomes in Patients With		CD-24 overexpression https://www.ncbi.nlm.nih.gov/pmc/articles/PMC10779124/	https://clinicaltrials.gov/study/NCT04747574	DRUG: EXO-CD24

	COVID-19 Infection				
NCT04664738	PEP on a Skin Graft Donor Site Wound	platelet-derived	Unmodified	https://clinicaltrials.gov/study/NCT04664738	BIOLOGICAL: 10% PEP丨DRUG: TISSEEL丨BIOLOGICAL: 20% PEP
NCT04602104	A Clinical Study of Mesenchymal Stem Cell Exosomes Nebulizer for the Treatment of ARDS	MSC		https://clinicaltrials.gov/study/NCT04602104	BIOLOGICAL: low dose hMSC-Exos丨BIOLOGICAL: medium dose hMSC-Exos丨BIOLOGICAL: high dose hMSC-Exos丨BIOLOGICAL: Dosage 1of hMSC-Exos丨BIOLOGICAL: Dosage 2 of hMSC-Exos丨BIOLOGICAL: No hMSC-derived exosomes
NCT04602442	Safety and Efficiency of Method of Exosome Inhalation in COVID-19 Associated Pneumonia	MSC		https://clinicaltrials.gov/study/NCT04602442	DRUG: EXO 1 inhalation丨DRUG: EXO 2 inhalation丨DRUG: Placebo inhalation
NCT04544215	A Clinical Study of Mesenchymal Progenitor Cell Exosomes Nebulizer for the Treatment of Pulmonary Infection	MSC		https://clinicaltrials.gov/study/NCT04544215	BIOLOGICAL: Dosage 1 of MPCs-derived exosomes丨BIOLOGICAL: Dosage 2 of MPCs-derived exosomes丨BIOLOGICAL: No MPCs-derived exosomes
NCT04493242	Extracellular Vesicle Infusion Treatment for COVID-19 Associated ARDS	MSC		https://clinicaltrials.gov/study/NCT04493242	BIOLOGICAL: ExoFlo丨OTHER: Intravenous normal saline

NCT04491240	Evaluation of Safety and Efficiency of Method of Exosome Inhalation in SARS-CoV-2 Associated Pneumonia.	MSC		https://clinicaltrials.gov/study/NCT04491240	DRUG: EXO 1 inhalation|DRUG: EXO 2 inhalation|DRUG: Placebo inhalation
NCT04388982	the Safety and the Efficacy Evaluation of Allogenic Adipose MSC-Exos in Patients With Alzheimer's Disease	MSC		https://clinicaltrials.gov/study/NCT04388982	BIOLOGICAL: low dosage MSCs-Exos administrated for nasal drip|BIOLOGICAL: mild dosage MSCs-Exos administrated for nasal drip|BIOLOGICAL: high dosage MSCs-Exos administrated for nasal drip
NCT04389385	COVID-19 Specific T Cell Derived Exosomes (CSTC-Exo)	T-cells		https://clinicaltrials.gov/study/NCT04389385	BIOLOGICAL: COVID-19 Specific T Cell derived exosomes (CSTC-Exo)
NCT04384445	Zofin (Organicell Flow) for Patients With COVID-19	Amnionic fluid		https://clinicaltrials.gov/study/NCT04384445	BIOLOGICAL: Zofin|OTHER: Placebo
NCT04356300	Exosome of Mesenchymal Stem Cells for Multiple Organ Dysfuntion Syndrome After Surgical Repaire of Acute Type A	MSC		https://clinicaltrials.gov/study/NCT04356300	BIOLOGICAL: Exosome of MSC

	Aortic Dissection				
NCT04313647	A Tolerance Clinical Study on Aerosol Inhalation of Mesenchymal Stem Cells Exosomes In Healthy Volunteers	MSC		https://clinicaltrials.gov/study/NCT04313647	BIOLOGICAL: 1X level of MSCs-Exo｜BIOLOGICAL: 2X level of MSCs-Exo｜BIOLOGICAL: 4X level of MSCs-Exo｜BIOLOGICAL: 6X level of MSCs-Exo｜BIOLOGICAL: 8X level of MSCs-Exo
NCT04276987	A Pilot Clinical Study on Inhalation of Mesenchymal Stem Cells Exosomes Treating Severe Novel Coronavirus Pneumonia	MSC		https://clinicaltrials.gov/study/NCT04276987	BIOLOGICAL: MSCs-derived exosomes
NCT04270006	Evaluation of Adipose Derived Stem Cells Exo.in Treatment of Periodontitis	adipose stem cells		https://clinicaltrials.gov/study/NCT04270006	BIOLOGICAL: adipose derived stem cells exosomes
NCT04213248	Effect of UMSCs Derived Exosomes on Dry Eye in Patients With cGVHD	MSC		https://clinicaltrials.gov/study/NCT04213248	DRUG: Umbilical Mesenchymal Stem Cells derived Exosomes
NCT04202770	"Focused Ultrasound and Exosomes to Treat	Amnionic fluid		https://clinicaltrials.gov/study/NCT04202770	OTHER: Exosomes

	Depression, Anxiety, and Dementias"				
NCT04202783	The Use of Exosomes In Craniofacial Neuralgia	Unspecified		https://clinicaltrials.gov/study/NCT04202783	OTHER: Exosomes
NCT04173650	MSC EVs in Dystrophic Epidermolysis Bullosa	MSC		https://clinicaltrials.gov/study/NCT04173650	DRUG: AGLE 102
NCT04134676	Therapeutic Potential of Stem Cell Conditioned Medium on Chronic Ulcer Wounds	MSC		https://clinicaltrials.gov/study/NCT04134676	DRUG: Conditioned Media
NCT03608631	iExosomes in Treating Participants With Metastatic Pancreas Cancer With KrasG12D Mutation	MSC		https://clinicaltrials.gov/study/NCT03608631	DRUG: Mesenchymal Stromal Cells-derived Exosomes with KRAS G12D siRNA
NCT03493984	Plant Exosomes and Patients Diagnosed With Polycystic Ovary Syndrome (PCOS) 17	Plant		https://clinicaltrials.gov/study/NCT03493984	OTHER: Ginger exosomes|OTHER: Aloe exosomes|OTHER: Placebo
NCT03437759	MSC-Exos Promote	MSC		https://clinicaltrials.gov/study/NCT03437759	BIOLOGICAL: exosomes derived from mesenchymal stem cells (MSC-Exo)

	Healing of MHs				
NCT03384433	Allogenic Mesenchymal Stem Cell Derived Exosome in Patients With Acute Ischemic Stroke	MSC		https://clinicaltrials.gov/study/NCT03384433	BIOLOGICAL: exosome
NCT02594345	Effect of Exosomes Derived From Red Blood Cell Units on Platelet Function and Blood Coagulation	Red Blood Cells		https://clinicaltrials.gov/study/NCT02594345	OTHER: in vitro study
NCT02565264	Effect of Plasma Derived Exosomes on Cutaneous Wound Healing	Plasma-derived exosomes		https://clinicaltrials.gov/study/NCT02565264	OTHER: plasma-derived exosomes
NCT02138331	Effect of Microvesicles and Exosomes Therapy on OI-cell Mass in Type I Diabetes Mellitus (T1DM)	MSC		https://clinicaltrials.gov/study/NCT02138331	BIOLOGICAL: MSC exosomes. Fentanyl patch
NCT01668849	Edible Plant Exosome Ability to Prevent Oral	Plant		https://clinicaltrials.gov/study/NCT01668849	"DIETARY_SUPPLEMENT: Grape extract|DRUG: Lortab mouthwash"

	Mucositis Associated With Chemoradiation Treatment of Head and Neck Cancer				
NCT01294072	Study Investigating the Ability of Plant Exosomes to Deliver Curcumin to Normal and Colon Cancer Tissue	Plant		https://clinicaltrials.gov/study/NCT01294072	DIETARY_SUPPLEMENT: curcumin│DIETARY_SUPPLEMENT: Curcumin conjugated with plant exosomes│OTHER: No intervention
NCT01159288	Trial of a Vaccination With Tumor Antigen-loaded Dendritic Cell-derived Exosomes	Dendritic cells		https://clinicaltrials.gov/study/NCT01159288	BIOLOGICAL: Dex2
NCT06495437	Preliminary Safety and Efficacy Study of Extracellular Vesicle Infusion in the Intervention of Age-related Phenotypes With Impaired Glucose Tolerance.	MSC		https://clinicaltrials.gov/study/NCT06495437	BIOLOGICAL: Intravenous infusion of extracellular vesicle preparation derived from Wharton's jelly mesenchymal stem cells.

NCT06253975	Randomized, Controlled, Multicenter Study of Extracellular Vesicles From Human Adipose Tissue Promoting Wound Healing	Adipose tissue		https://clinicaltrials.gov/study/NCT06253975	BIOLOGICAL: adipose tissue derived extracellular vesicles (AT-EVs)丨DRUG: Hyaluronic acid
NCT06242379	Safety and Efficacy of Stem Cell Small Extracellular Vesicles in Patients With Retinitis Pigmentosa	MSC		https://clinicaltrials.gov/study/NCT06242379	BIOLOGICAL: GMP compliant-BM-MSC derived sEVs
NCT06202547	Intra-ovarian Injection of MSC-EVs in Idiopathic Premature Ovarian Failure	MSC		https://clinicaltrials.gov/study/NCT06202547	BIOLOGICAL: Intra-ovarian injection of bone marrow mesenchymal stromal cells-derived extracellular vesicles
NCT06002841	Extracellular Vesicles From Mesenchymal Cells in the Treatment of Acute Respiratory Failure	MSC		https://clinicaltrials.gov/study/NCT06002841	BIOLOGICAL: intravenous treatment with EVs丨BIOLOGICAL: intravenous treatment with placebo solution
NCT05940610	The Safety and Efficacy of MSC-EVs in	MSC		https://clinicaltrials.gov/study/NCT05940610	BIOLOGICAL: MSC-EVs

	Acute/Acute-on-Chronic Liver Failure				
NCT05881668	MSC-EV in Acute-on-Chronic Liver Failure After Liver Transplantation	MSC		https://clinicaltrials.gov/study/NCT05881668	BIOLOGICAL: MSC-EV
NCT05836883	Study of ExoFlo for the Treatment of Perianal Fistulas	MSC		https://clinicaltrials.gov/study/NCT05836883	BIOLOGICAL: ExoFlo丨OTHER: Local injection of normal saline
NCT05774509	Treatment of Non-ischemic Cardiomyopathies by Intravenous Extracellular Vesicles of Cardiovascular Progenitor Cells	Cardiovascular progenitor cells		https://clinicaltrials.gov/study/NCT05774509	BIOLOGICAL: Extracellular vesicle-enriched secretome of cardiovascular progenitor cells differentiated from induced pluripotent stem cells
NCT05520125	Treatment of Patients With Bone Tissue Defects Using Mesenchymal Stem Cells Enriched by Extracellular Vesicles	MSC		https://clinicaltrials.gov/study/NCT05520125	BIOLOGICAL: Mesenchymal stem cells enriched by extracellular vesicles丨OTHER: Standard treatment of bone defects
NCT05176366	Study of ExoFlo for the Treatment of Medically	MSC		https://clinicaltrials.gov/study/NCT05176366	BIOLOGICAL: ExoFlo

	Refractory Ulcerative Colitis				
NCT05130983	Study of ExoFlo for the Treatment of Medically Refractory Crohn's Disease	MSC		https://clinicaltrials.gov/study/NCT05130983	BIOLOGICAL: ExoFlo
NCT05127122	Bone Marrow Mesenchymal Stem Cell Derived Extracellular Vesicles Infusion Treatment for ARDS	MSC		https://clinicaltrials.gov/study/NCT05127122	DRUG: Bone Marrow Mesenchymal Stem Cell Derived Extracellular Vesicles｜OTHER: Saline
NCT05125562	Extracellular Vesicles Infusion Treatment for Mild-to-Moderate COVID-19	MSC		https://clinicaltrials.gov/study/NCT05125562	DRUG: ExoFlo
NCT05116761	ExoFloтДв Infusion for Post-Acute COVID-19 and Chronic Post-COVID-19 Syndrome	MSC		https://clinicaltrials.gov/study/NCT05116761	BIOLOGICAL: Bone Marrow Mesenchymal Stem Cell Derived Extracellular Vesicles｜OTHER: Saline
NCT04761562	Use of Autologous Plasma Rich in Platelets and Extracellular	Plasma-derived exosomes		https://clinicaltrials.gov/study/NCT04761562	DRUG: Platelet- and extracellular vesicle-rich plasma｜PROCEDURE: Control group

	Vesicles in the Surgical Treatment of Chronic Middle Ear Infections				
NCT04698447	The Role of a Natural Product, Containing Nanovesicles From Citrus Limon (L.) Juice, on Different CV Risk Factors	Plant		https://clinicaltrials.gov/study/NCT04698447	DIETARY_SUPPLEMENT: Natural supplement containing nanovesicles delivered from Citrus Limon (L.) juice_MetS|DIETARY_SUPPLEMENT: Placebo (without any active ingredients)|DIETARY_SUPPLEMENT: Natural supplement containing nanovesicles delivered from Citrus Limon (L.) juice_HS
NCT04652531	Autologous Serum-derived EV for Venous Trophic Lesions Not Responsive to Conventional Treatments	Serum		https://clinicaltrials.gov/study/NCT04652531	OTHER: Autologous extracellular vesicles from serum
NCT04327635	Safety Evaluation of Intracoronary Infusion of Extracellular Vesicles in Patients Following Coronary Stent Implantation	blood		https://clinicaltrials.gov/study/NCT04327635	DRUG: PEP
NCT04281901	Efficacy of Platelet- and Extracellular Vesicle-rich	Plasma-derived exosomes		https://clinicaltrials.gov/study/NCT04281901	DRUG: Platelet- and extracellular vesicle-rich plasma|DRUG: Standard conservative treatment

	Plasma in Chronic Postsurgical Temporal Bone Inflammations				
